# An analysis of the transformative potential of Australia’s national food policies and policy actions to promote healthy and sustainable food systems

**DOI:** 10.1017/S1368980024000478

**Published:** 2024-02-20

**Authors:** Patricia Ribeiro de Melo, Phillip Baker, Priscila Pereira Machado, Elly Howse, Scott Slater, Mark Lawrence

**Affiliations:** 1 School of Exercise and Nutrition Sciences, Deakin University, Geelong, Australia; 2 The University of Sydney, School of Public Health, Sydney, Australia; 3 Institute for Physical Activity and Nutrition, Deakin University, Geelong, Australia; 4 NSW Government, Sydney, NSW, Australia

**Keywords:** Healthy and Sustainable Foods Systems, Food System Transformation and National Food Policy

## Abstract

**Objective:**

Despite commitment by many countries to promote food system transformation, Australia has yet to adopt a national food policy. This study aimed to evaluate Australian Federal Government’s (AFG) food policies and policy actions potential to promote healthy and sustainable food systems.

**Design:**

This study is a desk-based policy mapping followed by a theoretically guided evaluation of policy actions. This involved three steps: (1) identification of government departments and agencies that could influence Australia’s food system; (2) identification of food policies and policy actions within these departments and (3) use of a conceptual framework to evaluate policy actions’ potential of changing the food system as adjust (first-order change), reform (second-order change) or transform (third-order change).

**Setting:**

Australia.

**Participants:**

None.

**Results:**

Twenty-four food policies and sixty-two policy actions were identified across eight AFG departments and the Food Regulation System and evaluated based on the order of change they represented. Most policies were led by individual departments, reflecting the absence of a joined-up approach to food policy in Australia. Most policy actions (*n* 25/ 56·5 %) were evaluated as having adjust potential, whereas no transformative policy action was identified.

**Conclusions:**

These findings suggest that Australia is likely to proceed incrementally towards achieving food system change through adjustments and reforms but lacking transformative impact. To promote transformative change, all three orders of change must be strategically implemented in a coherent and coordinated matter. A comprehensive national food policy and a national coordinating body are needed to ensure a cohesive approach to policy.

Historically, food systems have been among the most significant environmental influences on the evolutionary trajectory of humans, nourishing and enabling thousands of generations to survive and thrive across the globe^(1)^. However, due to the current ways in which we produce and distribute foods, today’s food systems are neither healthy nor sustainable^(2,3)^. Recent estimates suggest that between 702 and 828 million people around the world are undernourished,^(3)^ while approximately 2 billion adults are overweight or obese^(4)^. In 2017, diet-related diseases accounted for approximately 11 million deaths around the globe^(5)^. Besides being a key contributor to adverse population health outcomes, food systems alone are responsible for nearly one-quarter of greenhouse gas emissions and around 40 % of the world’s habitable land usage, being therefore associated with multiple forms of environmental harms, such as deforestation, climate change and biodiversity loss^(6)^.

Recognising these challenges and acknowledging that achieving healthy and sustainable food systems is a critical step for the delivery of several of the UN Sustainable Development Goals, various expert groups and international organisations have called for a food system transformation^(7–9)^. Transformative solutions include those addressing food supply chains, food environments and consumer behaviour, while also emphasising the need for greater multi-sectorial work between all levels of governments and non-government institutions^(7–9)^. This is because food systems encompass a complex set of interlinked activities and processes which can generate multiple (and often conflicting) outcomes on a range of areas, such as health, agriculture, trade, transport, education and social security^(10)^. Converting potential policy tensions into leverage points for transformation will thus require the adoption of a comprehensive national food policy, a whole-of-government framework comprising a set of interventions that work synergically across multiple areas of the food system to promote healthy and sustainable population and planetary outcomes^(11)^. Notable examples of national food policies have been implemented in countries such as Finland^(12)^ Sweden^(13)^ and Canada^(14)^. It is important to note that these countries have been recognised for making significant advances towards the achievement of the Sustainable Development Goals^(15)^.

In Australia, the first and only National Food and Nutrition policy was implemented in 1992^(16)^. Despite influencing the development of federal and local level policies, this policy framework is no longer used to inform food-related policy activities in Australia^(17)^. In the absence of a national food policy, the National Preventive Health Strategy 2021–2030^(18)^ and the National Obesity Strategy 2022–2032^(19)^ are the two main nationwide strategies informing current actions to improve Australian population health outcomes. While addressing several health and nutrition-related concerns, these strategies display a set of stand-alone policy recommendations and therefore do not constitute a comprehensive approach to policy.

Several studies investigated food policies in Australia and their impacts on a range of health and/or sustainability outcomes. However, most of these were focused on examining government-led programs and initiatives within the Department of Health portfolio^(11,20,21)^. In an analysis conducted to investigate federally implemented nutrition policies led by the Australia’s Department of Health, a significant narrowing in the quantity and distribution of policy actions was identified. From 2007 to 2018, a notable shift from a coordinated approach to food policy towards a small number of disperse and modest policy actions (mostly with a focus on consumer behaviour change) was observed^(11)^. At local levels, Carrad and collaborators^(22)^ investigated Australia’s state jurisdictions responses to critical food systems issues led by different government departments and found that there was relevant state-level work being conducted to promote healthy and sustainable food systems.

Despite several studies examining food policy in Australia, further exploration of the transformative potential of federally implemented policies, beyond that of the Department of Health, is needed. To address this gap, this study aimed to investigate the transformative potential of AFG food policies and policy actions, within relevant government departments, to promote healthy and sustainable food systems. The definition of healthy and sustainable food systems used for this study is one that *‘ensures food security and nutrition for all in such a way that the economic, social and environmental bases to generate food security and nutrition of future generations are not compromised* ^(23)^.*’* Food policies are understood to be a statement of values, beliefs and intentions to shape the structure and/or operation of foods systems to promote health, nutrition and/or sustainability outcomes. For this study, policy actions are considered to be the means for translating policies’ recommendations into practical actions^(24)^. This research was structured around the following objectives: (1) to identify AFG departments and/or agencies whose roles and responsibilities could influence the structure and/or operation of Australia’s food system; (2) to identify food policies and policy actions within these departments and (3) to evaluate Australia’s food policies and policy actions’ potential for promoting transformative change in the food system.

## Methods

### Study design

This study consisted of a desk-based policy mapping followed by a theoretically guided evaluation of policy actions.

### Study setting and identification of the Australian Federal Government departments

Australia is a liberal democracy with a representative government system comprised of the federal Parliament, state, territory parliaments and local government. The AFG, also known as the Commonwealth Government of Australia, has three main institutions of power: the federal legislative government in the form of Parliament, the federal executive government and the judiciary^(11)^. Food policies and regulations are developed and implemented by different departments and statutory agencies, which are collectively known as the Australian Public Service^(11)^.

To identify AFG departments and statutory agencies that could have significant roles influencing the structure and/or operation of the food system, a cross-department mapping component adapted from the work developed by the Food Research Collaboration was conducted^(25)^. The AFG directory website was searched,^(26)^ and an investigation of Australian departments and their main food-related roles and responsibilities was conducted. A discussion among all team members occurred to achieve a consensus position on the selected departments.

### Identification of food policies and policy actions within Australian Federal Government departments and agencies

Most studies investigating food policies in Australia^(11,21,27)^ have focused on health department-initiated policies. This is understandable as historically in Australia, it has been the Department of Health which has explicitly taken primary responsibility for tackling nutrition and health problems^(16)^. As such, this analysis started with a focus on evaluating AFG food policies and policy actions within the Department of Health. A comprehensive search was conducted on the department’s website to identify current food policies and policy actions that could promote health, nutrition and/or sustainability outcomes. Once health-related policies were identified, this search was supplemented with food policies led by non-health departments. For these departments, a purposive sampling strategy was adopted for the identification of policies.

All departmental websites (health and non-health) were searched using keywords such as ‘food’ ‘nutrition’ ‘agriculture’ ‘diet,’ ‘health,’ ‘prevention’ ‘noncommunicable,’ ‘chronic,’ ‘obesity’ and ‘environment*.’ To ensure comprehensiveness in the investigation of the Department of Health initiated policies, an additional systematic search of the grey literature using the Google Advanced search tool was undertaken.

Food policies and policy actions were considered to be those that could influence the food system in terms of consumer behaviour, food environments and the food supply chain, by promoting health, nutrition and/or sustainability outcomes. Table 1 outlines the inclusion and exclusion criteria used for the identification of food policies and policy actions. The identification process was initially conducted by the lead author and subsequently reviewed by contributing authors. Where ambiguity existed, a discussion among all investigators occurred until consensus was achieved.

**Table 1:** Inclusion and exclusion criteria used for the identification of food policies and policy actions

Criterion	Inclusion	Exclusion
**Characteristics of the policy activities**	- Food policies or food-related regulatory standards that were active or in force at the time of the search. - Federal level food policies. - Policies that had a relevant policy role for shaping the structure and/or operation of food systems. - Policies related to the selected government departments.	- Policies that were no longer current or in effect at the time of the search. - State, territory and/ or local level policies. - Policies that did not have a relevant role for shaping the structure and/or operation of food systems, or which did not strive for change. - Policies that were outside the scope of the selected government departments.
**Institutions**	- Australian government organisations and/or partnerships and Australian federal government bodies.	- Non-governmental organisations/institutions.
**Types of policies**	- Legislations. - Decrees. - Acts. - Codes and other regulatory standards. - National policies. - Strategies, plans or frameworks and associated policy recommendations. - National based campaigns, programs and partnerships.	- Policy reference standards, such as dietary guidelines and NRVs. - Calls or proposals for policies which had not been agreed on by government bodies or policy submissions.

### Evaluation of food-related policy actions

Relevant documents were retrieved and information about food policies and policy actions was extracted and exported into an Excel spreadsheet. Food-related policy actions were evaluated against the Order of Food System Change schema, a conceptual framework developed by Lawrence and collaborators based on a review of the literature on systems change dynamics and practices^(28)^. This framework was selected given that its assessment criteria broadly reflect alternate views of policy actors towards the causes of and the solutions to food system-related challenges. Additionally, it has been previously used in studies investigating the transformative potential of food-related policy actions^(21)^ and global food policy recommendations^(29)^. The framework evaluates the ability of policies to achieve transformative change in the structure and/or operation of food systems by distinguishing them as either first-order change (adjust), second-order change (reform) or third-order change (transform), as outlined in Table 2. Broadly, first-order change policy actions are less disruptive of the system as they aim to adjust some of its isolated components. These include policies of labelling information or educational campaigns. Second-order change policy actions aim to improve the current system by reforming some of its structural and operational components. Examples include taxing unhealthy foods and international trade agreement to foods. Third-order change policy actions are the most disruptive of all policies as they aim to change the system’s entire orientation. An example of a third-order change approach is a comprehensive national food policy. Further details about the methodology used for the evaluation of policy actions can be found in Supplementary Text 1.

**Table 2:** Criterion for classifying food-related policy actions according to the orders of food system change conceptual framework*

Criterion	First-order change (Adjust)	Second-order change (Reform)	Third-order change (Transform)
*How the problem is framed, and its cause ascribed to the food system*	If a problem exists, it is a consequence of technical inefficiencies within the system design.	Accepts that there is a problem, and its cause(s) are associated with structural and operational shortcomings within the system.	Accepts the problem as a real and present danger and a consequence of a broken system created from flawed social, economic, and political values.
*Process for change*	Preserves the established power structure and relationships among actors in the system.	Challenges established power relationships shaping components within the system; promotes opportunities for interactions among a diverse range of actors in the system.	Promotes change in relationships towards whole-system awareness and identity; promotes examination of the deep structures that sustain the system.
*Government arrangements*	Projects within individual departments.	Programs across departments (usually led by health department).	Programs integrating all relevant departments (whole-of-government approach).
*Participation of stakeholders*	Replicates the established decision-making group and power relationships. Tends to be global in scope.	Brings relevant actors (government, civil society, academics and practitioners, producers, food industry) into the problem-solving conversation in ways that enable them to influence the decision-making process.	Promotes social inclusion, empowered producers and citizens actively engaged with the food system instead of being passive takers. Tends to be local in scope.
*Policy approach to bring about food system change*	Applies technological innovations to improve the resilience and/or adaptive capacity of components of the food system.	Applies a mechanistic analysis to identify leverage points within the system (distinct levels of government and/or sectors with responsibilities for system components) to reform their structure and operation.	Applies a system-level analysis to identify the system’s purpose and power relationships to reorientate its function from being predominantly a component of the industrialised economy to a health, social, environmental and economic resource.
*Examples of policies*	- Information campaigns. - Food labels. - Food reformulation. - Food fortification. - Nutrition education in schools.	- Subsidies to healthy foods or taxation of unhealthy foods. - Trade agreements to foods. - Regulatory approaches to food advertisement.	- A national comprehensive food policy or strategy. - A policy that promotes universal right to foods.

* Table adapted from^(28)^.

## Results

### Identification of Australian Federal Government departments’ roles and responsibilities that can influence the structure and/or operation of Australia’s national food system

**Fig. 1:**
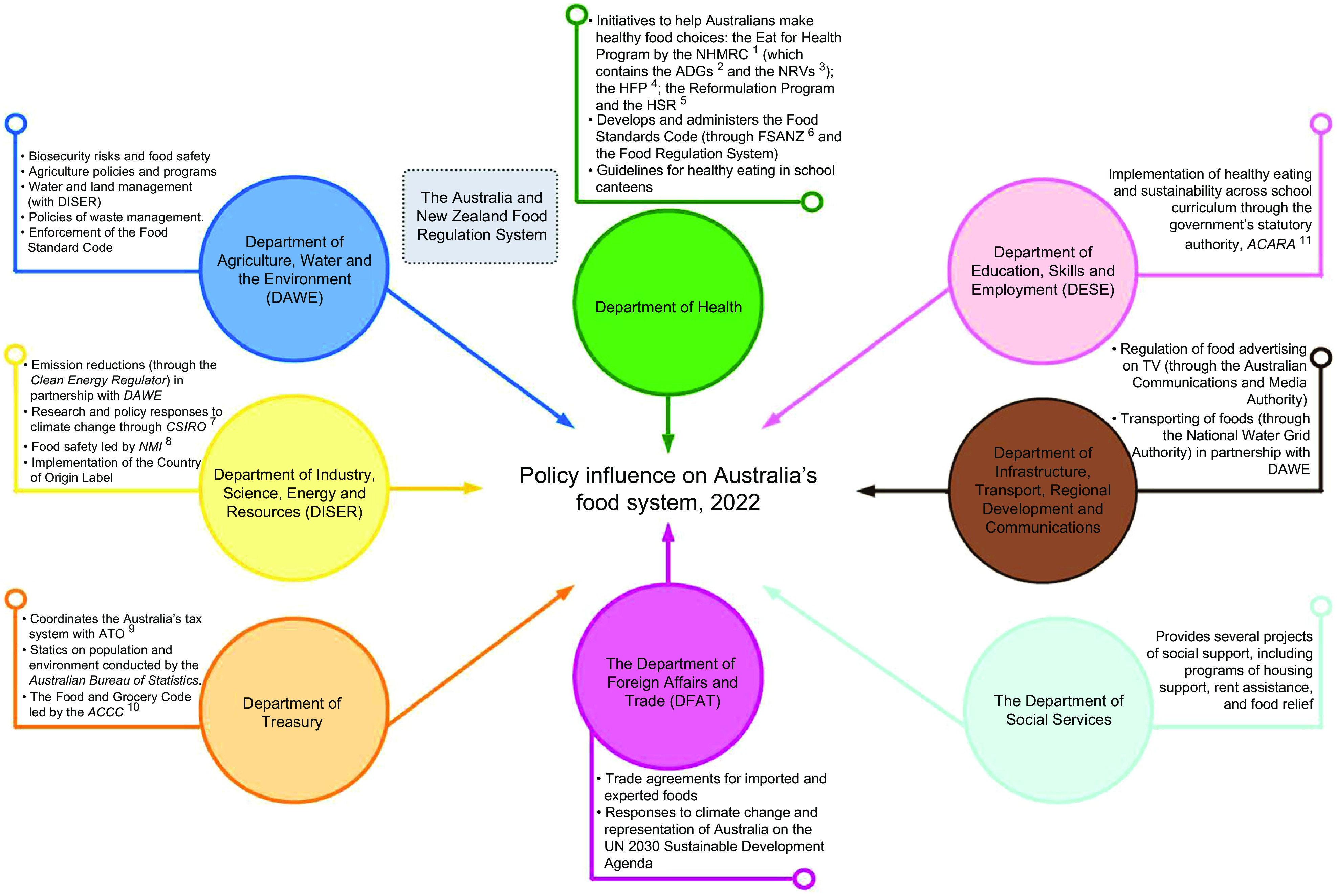
Australia’s federal government department’s policy roles and responsibilities that can influence the structure and operation of Australia’s food system. Abbreviations: ^1^NHMRC: The National Medical Research Council; ^2^ADGs: Australian Dietary Guidelines; ^3^NRVs: Nutrient Reference values; ^4^HFP: Healthy Food Partnership; ^5^HSR: Health Star Rating; ^6^FSANZ: Food Standards Australia New Zealand; ^7^CSIRO: Commonwealth Scientific and Industrial Research Organisation; ^8^NMI: National Measurement Institute; ^9^ATO: Australia Taxation Office; ^10^ACCC: Australian Competition and Consumer Commission; and ^11^ACARA: Australian Curriculum, Assessment and Reporting Authority. This diagram was inspired by the work developed by the Food Research Collaboration at the City University of London^25^

Eight AFG departments with significant roles influencing the structure and/or operation of Australia’s food system, and the Australia and New Zealand Food Regulation system, were identified. Figure 1 provides an overview of the departments’ main food policy-related roles and responsibilities.

The Department of Health’s food policy-related roles are the Eat for Health program, an initiative containing several resources to educate consumers on healthy eating; the Healthy School Canteens resource collection to set voluntary standards for the provision of foods and drinks supplied in school canteens and the Healthy Food Partnership, a voluntary partnership between governments and the food sector to support healthy eating. The Healthy Food Partnership contains four main working components of portion size, consumer education, food reformulation and the Health Start Rating, a nutrient profiling system that rates the healthiness of packaged foods and drinks^(30)^. Within the health portfolio is the Food Standards Australia New Zealand, a statutory authority that develops food standards in Australia. Through the Australia New Zealand Food Standards Code (the Code), Food Standards Australia New Zealand establishes foods standards for food labelling regulation, composition, production and safety. Compliance with the Code is enforced by state and territory authorities and, for imported foods, by the Department of Agriculture, Water, and the Environment (DAWE)^(31)^.

The National Health Medical Research Council is Australia’s main statutory authority conducting health and medical research and providing evidence-based advice to the community on a variety of health matters. This includes the development of a series of evidence-based guidelines for healthy eating (e.g. Australian Dietary Guidelines, 2013) and the Nutrient Reference Values for Australia and New Zealand^(32)^.

The Australia and New Zealand Food Regulation system, a cooperative joint system between the Australian and New Zealand governments, contains a range of policies and processes to ensure food safety of consumers in Australia. The system contains three components of (1) policy development: through which the Food Ministers’ Meeting and the Food Regulation Standing Committee develop food policy; (2) standard setting: Food Standards Australia New Zealand is responsible for developing, amending and setting food standards and (3) implementation and enforcement: Australian governments are responsible for the implementation, monitoring and enforcement of food regulation^(33)^.

DAWE’s main food policy-related roles are to facilitate sustainable agriculture and farming practices, promote sustainable management of natural resources, implement climate change mitigation strategies, support farmers in times of drought and hardship, oversee waste management policies, monitor biosecurity risks and ensure the safety of imported foods^(34)^.

The Department of Industry, Science, Energy and Resources (DISER) oversees policies of climate change and monitor businesses’ greenhouse gas emissions, energy production and consumption. Through the Commonwealth Scientific and Industrial Research Organization, the department carries out climate system research to inform climate change action. The National Measurement Institute, a peak measurement body within the DISER’s portfolio, works with the food sector to promote food safety and ensure appropriate food labelling^(35)^.

The Department of Treasury and the Australian Taxation Office administrate Australia’s taxation system to provide taxable rules for Australian services and goods, including foods. Within its portfolio is the Australian Bureau of Statistics, a statistical agency that collects population and environment-related data. Also within this department is the Australian Competition and Consumer Commission, a statutory authority that regulates the Food and Grocery Code to improve business conduct in the food and grocery sector. Treasury also provides financial advisory services for the development of major infrastructure projects across government departments, including food-related ones^(36)^.

The Department of Foreign Affairs and Trade facilitates Australia’s international relationships and support international trade and investment opportunities across the globe. Through the Australia’s free trade agreements, Department of Foreign Affairs and Trade establishes a set of international treaties between countries to facilitate the trade of goods, including imported foods. Department of Foreign Affairs and Trade also oversees Australia’s international response to climate change and represents the country on advancing the UN 2030 Agenda for Sustainable Development^(37)^.

The Department of Social Services aims to promote social security and improve population’s wellbeing through the delivery of a variety of projects of housing support, rent assistance, income support payment and food relief programs^(38)^.

The Department of Infrastructure, Transport, Regional Development and Communications and Industry main food policy-related roles are to facilitate the transporting of foods and improve access to water for farming practices across Australia. Through the Australian Communications and Media Authority, the department regulates free-to-air television advertisement, which includes setting rules for food marketing during children’s programming^(39)^.

The Department of Education, Skills, and Employment and the Australian Curriculum, Assessment and Reporting Authority ensure nutrition education within schools to students by integrating the topic of healthy eating across specific learning areas outlined in the Australian School Curriculum. Sustainability is also one of three cross-curriculum priorities, being addressed in all seven curriculum learning areas^(40)^.

### Identification of food policies within Australian Federal Government departments and evaluation of policy actions

A total of twenty-four policies were identified, and sixty-two policy actions were evaluated based on their transformative potential. As listed in see online supplementary material, Supplementary Table 1, most of the assessed policy actions (*n* 39) were led by the Department of Health, followed by DAWE (*n* 9), DISER (*n* 4), the Department of Infrastructure, Transport, Regional Development and Communications (*n* 4), the Department of Social Services (*n* 3), Treasury (*n* 1), Department of Foreign Affairs and Trade (*n* 1) and Department of Education, Skills, and Employment (*n* 1). Most of the Department of Health policies targeted food environments (45·9 %) and consumer behaviour (37·9 %). Out of the twenty-four policy actions that targeted the food supply chains, eighteen were led by non-health departments. Information about food policies and policy actions can be found in Table 3. See online supplementary material, Supplementary Table 2 contains a more detailed description of food policies and policy actions.

**Table 3:** Identification of Australian Federal Government food policies and classification of policy actions according to the orders of change they represent

Responsible federal government departments	Food policies	Policy actions	Current status	Order of change	Food system focus
**The Department of Health**					
The Department of Health	*Get Up & Grow – Healthy eating and physical activity for early childhood*	Provision of nutrition guidance through information campaigns and resources.	Available to the public.	First-order change	Consumer behaviour
The Department of Health (through the National Health and Medical Research Council’s – NHMRC)	*The Eat for Health website*	Public health nutrition guidelines and consumer information resources for healthy eating.	Available to the public.	First-order change	Consumer behaviour
The Department of Health	*feedAustralia*	Nutrition guidance through the online feedAustralia App.	Available to the public.	First-order change	Consumer behaviour
The Department of Health	*The National Healthy School Canteens (NHSC) project*	Guidelines to help canteen managers make informed assessments of the nutritional value of foods supplied in school canteens.	Available to the public. Guidelines and resources are voluntary and do not provide endorsement of food or drink products.	Second-order change	Food environment
The Department of Health	*Healthy Food Partnership (HFP)*	The Health Star Rating (HSR) system to assess the ‘healthiness’ of packaged food products based on their nutrient profile.	Active (voluntary).	First-order change	Food environment
		Voluntary Food Reformulation Program to reduce sugar, sodium and saturated fat in processed and manufactured food and drinks.	Active (voluntary).	First-order change	Food supply chain
		The Industry Guide to Voluntary Serving Size Reduction: encourages food manufacturers to promote appropriate serving sizes.	Available to the public. This guideline is voluntary and does not provide endorsement of serving sizes.	First-order change	Food supply chain
		Provision of nutrition guidance to educate Australians on healthy eating.	No further information is available.	First-order change	Consumer behaviour
The Department of Health	National Preventive Health Strategy (NPHS) (2021–2030)	NPHS supports two policy actions recommending that (i) nutrition and food action in Australia is guided by a specific national policy document and (ii) a national policy document is developed to address food security in priority populations.	Policy achievements to be attained by 2030.	Second-order change	Unclear
		NPHS supports four policy actions of translation and widespread promotion of nutrition information and guidance to the public.	Policy achievements to be achieved by 2030.	First-order change	Consumer behaviour
		NPHS supports one policy action of co-designed, community-based programs to meet the nutritional needs of priority populations.	Policy achievement to be attained by 2030.	Second-order change	Consumer behaviour
		NPHS supports two policy actions to improve the quality of the food supply chain: the Food Reformulation Program, and initiatives of serving size reduction.	Policy achievements to be attained by 2030.	First-order change	Food supply chain
		NPHS supports two policy actions recommending that consumer choice is guided by energy and ingredient labelling as well as the Health Star Rating system.	Policy achievements to be attained by 2030.	First-order change	Food environment
		NPHS supports five policy actions to improve the quality of food environments, which includes (i) restricting food advertisement and promotion of unhealthy food products and increasing the promotion of and accessibility to healthy food options; (ii) addressing the structural and environmental barriers to breastfeeding and (iii) use of economic tools to facilitate healthier consumer behaviour (e.g. tax reform).	Policy achievements to be attained by 2030.	Second-order change	Food environment
Commonwealth of Australia as represented by the Health Ministers Meeting 2022	*National Obesity Strategy (NOS) (2022–2032)* *Enabling Australians to eat well and be active*	NOS supports one policy action to facilitate food systems that favour the production, processing and distribution of healthy food and drinks (e.g. food trade agreements to support healthier food supply chains).	Policy strategy to be attained by 2032.	Second-order change	Food supply chain
		NOS supports three policy actions to improve food environments and help consumers make healthier food choices through land use planning schemes, use of economic tools to shift consumer purchases towards healthier foods and reducing exposure to unhealthy food marketing.	Policy strategies to be attained by 2032.	Second-order change	Food environment
		NOS supports one policy action to make processed foods and drinks healthier through initiatives of food reformulation and serving sizes reduction.	Policy strategy to be attained by 2032.	First-order change	Food supply chain
		NOS supports two policy actions to improve nutrition information to help consumers make healthier choices.	Policy strategies to be attained by 2032.	First-order change	Food environment
		NOS supports two policy actions to enable education settings and workplaces to better support the health and wellbeing of children, young people and employees.	Policy strategies to be attained by 2032.	Second-order change	Food environment
		NOS supports five policy actions to improve people’s knowledge and skills to help them make healthier food choices through nutrition education, social marketing and engagement with local communities.	Policy strategies to be attained by 2032.	First-order change	Consumer behaviour
Food Standards Australia New Zealand – FSANZ, the Food Regulatory System, and DAWE (responsible for the enforcement of the code)	*The Australia New Zealand Food Standards Code, 1991*	Sets legal standards for composition of food products, food labelling, food safety and production of foods in Australia.	In force	Second-order change	Food environment
**The Department of Agriculture, Water and the Environment (DAWE)**					
DAWE and DISER	*The National Landcare Program (NLP)*	The Regional Land Partnerships (RLP): nationwide projects to contribute to the recovery of threatened species and protection of ecological communities through investments in land care.	Active	Second-order change	Food supply chain
		Environment Small Grants (ESG): provides funding for local community projects to protect and conserve Australia’s water, plants and animals and the ecosystems.	Active	First-order change	Food supply chain
		Regional Natural Resource Management (NRM): delivery agents under the regional stream of the NLP.	Active	First-order change	Food supply chain
		Landcare Networks: networks responsible for information sharing and coordination of the issues facing on-ground volunteers.	Active	First-order change	Food supply chain
DAWE	*Drought Policy*	The Australian Government works with all involved as they prepare for, manage and recover from drought.	Active	Second-order change	Food supply chain
DAWE	*The Australian Food Pact*	A voluntary agreement that encourages organizations to develop tailored food waste action plans focusing on reducing food waste.	Active (voluntary agreement)	Second-order change	Food supply chain
DAWE	*The Agriculture Biodiversity Stewardship Package*	Provision of rewards to landholders for undertaking carbon plantings, increasing biodiversity and retaining and improving existing native vegetation on privately owned lands.	On trial (phase one)	First-order change	Food supply chain
		The voluntary Australian Farm Biodiversity Certification Scheme: a voluntary certification scheme that enables consumers to identify farms that sustain biodiversity.	Active (voluntary certification)	First-order change	Consumer behaviour
		The National Stewardship Trading Platform: a platform that integrates spatial information alongside buyer and seller to enable landholders to connect with buyers of biodiversity outcomes.	Active	First-order change	Food supply chain
**The Department of Industry, Science, Energy and Resources (DISER)**					
DISER and Treasury	*Country of Origin Food Labelling Information Standard 2016 (Cth)*	Helps consumers make informed decisions about where food they buy is grown, produced, made or packaged.	In force	First-order change	Food environment
DISER	*Climate Active*	A Climate Active certification is awarded to organisations products, services, events, precincts and buildings that have credibly reached a state of carbon neutrality.	Active (voluntary partnership)	First-order change	Food supply chain
DISER (through the Clean Energy Regulator)	*The National Greenhouse and Energy Reporting Act 2007 (NGER Act) and the National Greenhouse and Energy Reporting (NGER) scheme*	The NGER Act introduced the NGER scheme for reporting and disseminating company information about greenhouse gas emissions, energy production and energy consumption and other information.	In force	Second-order change	Food supply chain
DISER (through the Clean Energy Regulator) and DAWE	*The Emissions Reduction Fund (ERF)*	ERF offers landholders, communities and businesses the opportunity to run projects in Australia that avoid the release of greenhouse gas emissions or remove and sequester carbon from the atmosphere.	Enacted through the Carbon Credits Act 2011 and the Carbon Credits Rule 2015	Second-order change	Food supply chain
**Treasury**					
*Australian Taxation Office (ATO)*	*Goods and services tax (GST) – Section 38*-2 *of the Act 1999*	Provides taxable rules for foods and exempts certain staple foods from being taxed.	In force (the GST on foods was introduced in July 2000)	Second-order change	Food environment
**The Department of Foreign Affairs and Trade (DFAT)**					
DFAT and DAWE	*Australia’s free trade agreements (FTA) for foods*	Reduces and eliminate certain barriers to international trade and allow Australian exporters, importers and producers to expand their business into foreign markets.	In force	Second-order change	Food supply chain
**The Department of Social Services**					
Department of Social Services (DSS)	*Emergency Relief National Coordination Plan*	Emergency Relief and Food Relief Support: through these services, the government aims to increase Emergency Relief providers’ access to a cost-effective supply of food items, across Australia.	Active	First-order change	Food supply chain
		Supporting service providers in responding to the coronavirus outbreak: the Australian Government is supporting service providers in responding to the coronavirus outbreak, as well as directing funding for Emergency Relief and Food Relief support.	On 29 March 2020, the Australian Government announced funding to support charities and other community organisations in responding to the coronavirus outbreak.	First-order change	Food supply chain
		The National Coordination Plan: the national coordination plan supports the identification and analysis of local, state and sector issues and needs and oversees the implementation of emergency and food relief across the country.	Active	First-order change	Food supply chain
**The Department of Infrastructure, Transport, Regional Development and Communications**					
Department of Infrastructure, Transport, Regional Development and Communications (through the Australian Communications and Media Authority)	*Broadcasting Services Act 1992 (the Act)*	The Act contains one provision that specifically restricts unhealthy food advertising during children programming as well as other general rules on advertising which are also relevant to foods.	In force	Second-order change	Food environment
The Department of Infrastructure, Transport, Regional Development and Communications and DAWE	*Regional Airports Program (RAP)*	A program that facilitates improved delivery of essential goods and services such as food supplies, health care and passenger air services.	Active	Second-order change	Food supply chain
Department of Infrastructure, Transport, Regional Development and Communications (through the National Water Grid Authority)	*Australia’s National Water Grid*	A series of region-specific water storage and distribution solutions to secure predictable supplies of water now and into the future.	Active	Second-order change	Food supply chain
The Department of Infrastructure, Transport, Regional Development and Communications and DAWE	*Inland Rail*	An infrastructure project that will facilitate the transporting the food from agricultural land Australia produces to domestic and international communities.	Underway	Second-order change	Food supply chain
**The Department of Education, Skills and Employment (DESE)**					
The Australian Curriculum, Assessment and Reporting Authority	*The Australian National Curriculum*	Addresses student learning regarding food and nutrition through the Health and Physical Education subject.	Implemented (ongoing)	First-order change	Consumer behaviour

Nearly 60 % of the Department of Health policy actions have first-order change potential, mostly consisting of educational campaigns and resources, food labelling regulation, food reformulation and portion size standardisation. Out of the sixteen s-order change policy actions, the National Healthy School Canteens and the Code were the only implemented policies. The remaining second-order change health-related policy actions were recommendations derived from the National Preventive Health Strategy and National Obesity Strategy, which mostly focused on food marketing regulation, use of economic tools to shift consumer behaviour towards healthier eating, interventions to improve food accessibility and affordability, trade agreements to foods and policies of food procurement.

Out of the twenty-three policy actions led by non-health departments, twelve were first-order potential change and eleven second-order potential. The first-order change policy actions included those of food labelling, incentives for carbon emission reduction, projects of social support and food relief and interventions of nutrition education in schools. Non-health policy actions evaluated as second-order change were those of regional land partnerships, drought support to farmers, management of food waste, taxable rules for foods, international trade agreements on foods, marketing restrictions on unhealthy foods, transporting of foods, water storage and distribution and monitoring and/or reduction of gas and carbon emissions.

Most policies identified in this study (*n* 17) were led by individual departments. The Department of Health, the Australia and New Zealand Food Regulatory System, DAWE, DISER, Treasury and The Department of Infrastructure, Transport, Regional Development and Communications were the government sectors which demonstrated a deliberate cross-departmental collaboration with the delivery and enforcement of the Code, The National Landcare Program, the Country of Origin Food Labelling, the Emissions Reduction Fund, trade agreements for foods, the Regional Airports Program and the Inland Rail.

**Fig. 2:**
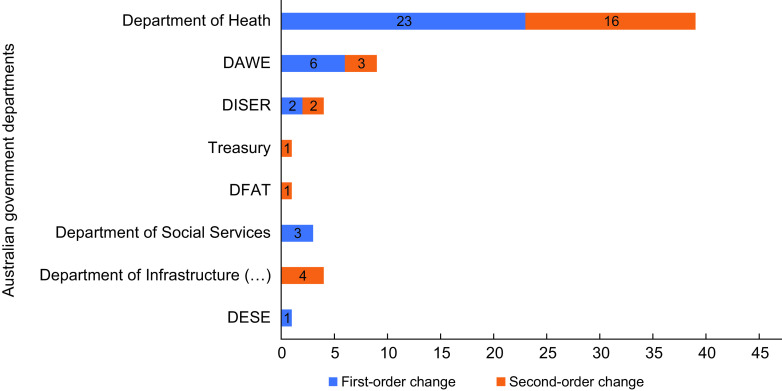
Distribution of food-related policy actions across the Australian Federal Government departments and according to the order of change they represent. Abbreviations: DAWE: Department of Agriculture, Water and the Environment; DISER: Department of Industry, Science, Energy and Resources; DFAT: Department of Foreign Affairs and Trade; Department of Infrastructure (…): Department of Infrastructure, Transport, Regional Development and Communications; DESE: Department of Education, Skills and Employment

In total, twenty-five policy actions (56·5 %) were evaluated as first-order potential, and twenty-seven (43·5 %) as second-order change potential. No third-order potential policy action was identified in this analysis. Figure 2 shows the cross-departmental distribution of policy actions according to the order of change they represent. The Department of Health was leading most of the first-order change policy actions (*n* 23) identified in this analysis. While a mixed distribution of orders of change was noted in the departments of Health, DAWE and DISER, the remaining departments were leading either first- or second-order change policy actions only.

## Discussion

This study aimed to identify AFG departments and associated agencies that could influence the structure and/or operation of Australia’s food system, identify their main food policies and policy actions and evaluate their transformative potential for promoting healthy and sustainable food systems. Broadly, four major findings emerged from this analysis. First, there has been a tendency for siloed policy work to address food-related concerns in Australia, as most of the policies identified in this analysis were led by individual departments. Second, a joined-up approach to food policy was not evident among AFG departments, given that there was no indication of linkage between food policy-related roles and responsibilities. Siloed work in food policy has been previously reported in Australia^(41)^ as well as in other countries^(25)^. Fragmented policymaking can lead to an incoherent policy environment, whereby government departments pursue mutually exclusive goals, and policies often contradict one another^(42)^. In practice, this could translate into one department supporting a reduction in livestock production for environmental purposes, whereas another department would be pursuing trade deals to increase the availability of animal-based food products in the supply chain^(42)^.

The third main finding of this analysis relates to the differences between departments in terms of number of policies and their potential for promoting food system change. Most policy actions identified in this analysis were those led by the Department of Health, most of which were evaluated as first-order potential. Consistent with its main food-related goal, which is to help Australians make healthier food choices^(30)^, health-initiated food policies and policy actions had significant focus on supporting individual behavioural change, mainly through approaches of food labelling and awareness-raising campaigns. Another important policy action within this department was the Voluntary Food Reformulation Program. Not only was this initiative currently implemented under the Healthy Food Partnership portfolio, but also receiving support from two national health strategies (National Obesity Strategy and National Preventive Health Strategy)^(18,19)^. Non-health departments, on the other hand, were found to have greater scope for leading reformative potential policy actions when compared to the Department of Health. This could be because their policies are mostly focused on promoting structural changes to food supply chains and food environments. Examples include policies of free trade agreements to foods, infrastructure projects to facilitate the transporting of water and foods across the supply chain, policies of waste management and interventions that exempts staple foods from being taxed (the GST system). These findings tell us that while the Department of Health may be the one leading the highest number of food-related policy actions in Australia, it might not be the best placed sector for promoting transformative change. In reality, all government departments have a role to play in transforming the food system as they each can contribute to the areas for which they are responsible. Hence, it is the combination of the amount, as well the strategic variety of orders of change across departments, which will determine the overall potential of a food system to be transformed.

The fourth main finding of this analysis relates to Australia’s overall response to systemic food system challenges, which is mostly composed of first-order change policy actions, followed by second-order change ones. No third-order potential policy action was identified in this analysis, which indicates that progress towards food system change in Australia is likely to lack transformative impact. These findings corroborate with that reported in the literature. In a study that assessed the scope of national nutrition policies for achieving food system transformation in high-income countries, a tendency towards behavioural change policies (e.g. information campaigns), or technological fixes in the food supply (e.g. food reformulation), was identified in most countries, including Australia^(27)^. In recent studies in which the Order of Food System change schema was used to evaluate the transformative impact of global food system report recommendations^(29)^ and policy actions to reduce added sugar consumption in Australia^(21)^, third-order change recommendations were found to be significantly low^(29)^ or non-existent^(21)^. The main reasons for the low transformative impact reported in these studies were the presence of stakeholders with vested interests in the decision-making processes^(29)^, the dominance of neoliberal ideologies focused on individual responsibility^(27)^, fragmentation of public health stakeholders and policymakers’ unwillingness to promote change^(21)^.

There are several explanations that may help understand Australia’s slow progress towards the achievement of transformative food system change. One such explanation is the complex and interconnected nature of food systems. Food systems are dynamic systems that encompass a variety of activities, actors, contexts and drivers^(23)^. Each of these elements has multiple inputs and outputs, as well as interacting components which create complex dynamics of balancing and reinforcing feedback loops throughout the system^(22)^. These characteristics mean that intervening in one part of the system can generate far-reaching effects and unintended consequences elsewhere. For instance, while increasing automation of food production may help decrease the purchasing costs of foods, this may also contribute towards increased energy use and decreased jobs in agriculture regions^(42)^. Because first-order change solutions are less disruptive of the system, they tend to be easily assimilated by decision-makers. Conversely, transformative policies that imply a whole-of-system change can often be perceived as ‘resource-intensive’ and faced with resistance by policy actors^(28)^.

Another potential explanation for the low transformative potential of Australia’s food policies is the influential power of actors with vested interests in the decision-making process. In Australia, food policy sits within the remit of federal and local governments^(11)^, but the overall decision-making process can be influenced by several external actors^(43)^. An analysis that investigated the level of influence of policy actors over nutrition policymaking in Australia found that, when compared to other stakeholder groups, such as non-government organisations, academics and the media, the food industry had greater access to key government members, allowing them with significant power to influence policy^(43)^. The problem with securing such a privileged position in the decision-making process is that, in furthering their own interests, these actors can institutionalise and perpetuate systemic and self-reinforcing dynamics which prevent the system from shifting its current trajectory. Similarly, the political interests of representatives of the public sector can, at times, be intertwined with those of the private sector, limiting their ability to progress change^(44)^. The power of vested interests has proven to obstruct meaningful food system change in Australia by, for example, hindering the integration of sustainability considerations into dietary guidelines^(45)^.

Lastly, conflicting views on how to address food-related problems, even amongst key public health groups or government sectors, may hinder policy actors’ ability to collaborate towards promoting meaningful change^(44)^. For example, progress on developing an integrated food and nutrition policy in Australia was sidelined in 2013 by, among other reasons, divergent views of key sectors regarding whether the focus of the plan should be on domestic food supplies or food exports^(45)^.

It has been argued that Australia’s socio-political context, and its reliance on a production-oriented model focused on agricultural productivity and trade liberalisation, is not ‘sufficiently mature’ to generate transformative change^(45)^. Recently, this assertion became particularly evident when, in response to the UN Food Systems Summit hosted in 2021, the Ministry of Agriculture announced Australia’s intent of investing in innovative technologies to improve agricultural systems. However, the statement was narrowly focused on food production and did not mention the need to promote healthier and more sustainable food systems^(46)^.

The pathway to achieve food system transformation in Australia will require a coherent combination of all three levels of order of change^(28)^. For instance, first-order change policy actions to guide individual behaviour (e.g. food labelling) could be reinforced through the implementation of second-order change interventions, such as those of market-based subsidies to support consumers in making healthier food choices. These strategies could in turn be backed up by a comprehensive whole-of-government framework to strategically combine several interventions across government departments and policy areas, while creating opportunities for different policies to support one another. One powerful example of a strategic combination of multi-level and multi-sectoral policies to promote health and sustainability outcomes is the Brazil’s Zero Hunger Program, a comprehensive nationwide strategy regarded as an international benchmark for addressing poverty, food insecurity and promoting rural development. Launched in 2003, the program delivered a set of synchronised strategies aimed at eradicating hunger and extreme poverty, which included: a nationwide cash transfer program that benefited over 11 million families, initiatives of rural credits to subsidy family farming, the strengthening of the national school meal program and policies of institutional food procurement to support smallholder farmers^(47)^. As a result of this synergetic combination of actions, the rate of extreme poverty reached its lowest historical level of 3·4 % in 2014, the income of smallholder farmers increased by 33 % between 2003 and 2008 and the amount of undernourished people dropped by over 80 % within a 24-year period^(48)^. The outstanding success of the program was, among other reasons, attributed to its coordinated and participatory approach to addressing food insecurity characterised by the establishment of a National Food and Nutrition Security Council and the consolidation of a National Food and Nutrition Security policy and system^(47)^.

Presently, there is no coordinated approach to food policy, or a specific government body responsible for overseeing cross-sectoral responses to systemic food system challenges in Australia. Despite one recommendation outlined in the landmark ‘Labelling Logic’ report in 2011, regarding the development of a national food policy^(49)^, this was overlooked for other first-order change initiatives, such as those led by the Healthy Food Partnership ^(11)^. Encouragingly, the AFG announced in 2022 an investment of $700 000 towards developing scoping review to inform the making of a National Nutrition Policy Framework. The findings from this scoping review are yet to be released. An effective outcome would be for this review to highlight the need for a joined-up and multi-sectorial approach to food policy with a focus on protecting and promoting both population and planetary health^(50)^.

### Limitations

To the best of the authors’ knowledge, this is the first study to evaluate AFG food policies and policy actions’ potential to change the structure and/or operation of food systems. Despite its novel contribution to the field, some limitations should be acknowledged to contextualise the interpretation of the results and guide future research endeavours.

First, the use of the Order of Food System Change framework introduces an inherent limitation to the analysis, as its assessment criteria are subject to interpretation. Policies may have components that span multiple categories within the framework, which could potentially generate a certain level of ambiguity during the evaluation process. To mitigate this, a three-step approach to policy evaluation was adopted, as described in Supplementary Text 2.

Another limitation is that this study was focused on evaluating the transformative potential of policy actions, as opposed to their current contribution to changing Australia’s food system. Consequently, specific design and implementation characteristics of policies were not investigated in this analysis, which may have limited a comprehensive understanding of their immediate impact to food systems. Similarly, this study was limited to evaluating federal level policies only, meaning that potentially transformative policies implemented at other government levels were not included in this analysis. For a more comprehensive evaluation of Australia’s food policy landscape, future studies should consider analysing the transformative impact of food policies at various government levels.

Lastly, a purposive sampling strategy was employed for the selection of non-health department led policies. As a result, it is likely that not all existing food policies and policy actions were included for this analysis. Moving forward, a more in-depth investigation of non-health food policies is needed.

### Conclusion

Currently, Australia lacks a comprehensive national food policy. Instead, there are several first- and second-order change policy actions underway to address some food-related challenges, most of which are being implemented in a fragmented and uncoordinated manner by different government departments. The absence of third-order change policy actions and the lack of a coherent and joined-up approach to policy means that progress towards achieving food system change in Australia is likely to proceed incrementally through adjustments and some reforms, but lacking transformative impact to challenge fundamental structures that sustain the current system. To tackle systemic food system challenges, all three orders of change need to be strategically combined and translated into policy actions in a coherent and coordinated matter. In practice, this means broadening the scope of the current food policy agenda from being predominantly focused on a constrained range of isolated policy actions, towards tackling the social, commercial and political determinants of health. A comprehensive national food policy, alongside the consolidation of a cross-government coordinating body, is urgently needed to ensure a cohesive response to food system challenges. Moving forward, more investments in third-order change policies are necessary to help shift the structure and operation of food systems away from unhealthy and unsustainable outcomes and towards the achievement of the Sustainable Development Goals.

## Supporting information

Ribeiro de Melo et al. supplementary material 1 Ribeiro de Melo et al. supplementary material

Ribeiro de Melo et al. supplementary material 2 Ribeiro de Melo et al. supplementary material

Ribeiro de Melo et al. supplementary material 3 Ribeiro de Melo et al. supplementary material

## Supplementary material

For supplementary material accompanying this paper visit https://doi.org/10.1017/S1368980024000478
