# Supplementary material for: An analysis of the transformative potential of Australia’s national food policies and policy actions to promote healthy and sustainable food systems
Source: Public Health Nutr. 2024 Feb 20;27(1):e75. doi: 10.1017/S1368980024000478 (PMC10966843; doi:10.1017/S1368980024000478)
Supplement: Ribeiro de Melo et al. supplementary material 1 — Ribeiro de Melo et al. supplementary material [file S1368980024000478sup001.docx]

**Supplementary Table 1.** Distribution of Australian Federal Government food policies and policy actions according to the orders of change they represent

| **Government Departments** | **Policies** | | | | **Order of change classification** | | | | | | **Food system focus** | | | | | |
| --- | --- | --- | --- | --- | --- | --- | --- | --- | --- | --- | --- | --- | --- | --- | --- | --- |
|  | *Food policies* | | *Policy actions* | | *First-order change* | | *Second-order change* | | *Third-order change* | | *Consumer behavior* | | *Food environment* | | *Food supply chain* | |
|  | **n** | **%** | **n** | **%** | **n** | **%** | **n** | **%** | **n** | **%** | **n** | **%** | **n** | **%** | **n** | **%** |
| The Department of Health | 8 | 33.4 | 39 | 62.9 | 23 | 59.0 | 16 | 41.0 | 0 | 0.0 | 14 | 37.9 | 17 | 45.9 | 6 | 16.2 |
| The Department of Agriculture, Water, and the Environment (DAWE) | 4 | 16.6 | 9 | 14.5 | 6 | 66.7 | 3 | 33.3 | 0 | 0.0 | 1 | 11.1 | 0 | 0 | 8 | 88.9 |
| The Department of Industry, Science, Energy and Resources (DISER) | 4 | 16.6 | 4 | 6.5 | 2 | 50.0 | 2 | 50.0 | 0 | 0.0 | 0 | 0.0 | 1 | 25.0 | 3 | 75.0 |
| Treasury | 1 | 4.2 | 1 | 1.6 | 0 | 0 | 1 | 100.0 | 0 | 0.0 | 0 | 0.0 | 1 | 100.0 | 0 | 0 |
| The Department of Foreign Affairs and Trade (DFAT) | 1 | 4.2 | 1 | 1.6 | 0 | 0 | 1 | 100.0 | 0 | 0.0 | 0 | 0.0 | 0 | 0.0 | 1 | 100.0 |
| The Department of Social Services | 1 | 4.2 | 3 | 4.8 | 3 | 1.6 | 0 | 0.0 | 0 | 0.0 | 0 | 0.0 | 0 | 0.0 | 3 | 100.0 |
| The Department of Infrastructure, Transport, Regional Development and Communications | 4 | 16.6 | 4 | 6.5 | 0 | 0.0 | 4 | 100.0 | 0 | 0.0 | 0 | 0.0 | 1 | 25.0 | 3 | 75.0 |
| The Department of Education, Skills, and Employment (DESE) | 1 | 4.2 | 1 | 1.6 | 1 | 100.0 | 0 | 0.0 | 0 | 0.0 | 1 | 100.0 | 0 | 0.0 | 0 | 0.0 |
|  | **TOTAL** | | | | | | | | | | | | | | | |
|  | 24 | 100.0 | 62 | 100.0 | 35 | 56.5 | 27 | 43.5 | 0 | 0.0 | 16 | 26.7 | 20 | 33.33 | 24 | 40.0 |
