# Supplementary material for: An analysis of the transformative potential of Australia’s national food policies and policy actions to promote healthy and sustainable food systems
Source: Public Health Nutr. 2024 Feb 20;27(1):e75. doi: 10.1017/S1368980024000478 (PMC10966843; doi:10.1017/S1368980024000478)
Supplement: Ribeiro de Melo et al. supplementary material 2 — Ribeiro de Melo et al. supplementary material [file S1368980024000478sup002.docx]

## **Supplementary Text 1**

## *Guidelines used for the evaluation of food-related policy actions*

To ensure a consistent methodology for the evaluation of policy actions and reduce subjectiveness, the authors conducted a three-step approach to the analysis which involved: (1) Initial classification of policy actions against the Order of Food System Change conceptual framework by all authors (PB, PM, EH, SS, and ML); (2) Discussion of the classification of policy actions to achieve a consensus when the authors had different assessments (PR, PB, PM, EH, SS, and ML); and (3) Final classification of policy actions by the lead author (PR). The classification assessment was informed by four guidelines:

1. The **ability** of a food-related policy action to change the structure and/or operation of the food system. This means that the evaluation of the extent to which a policy action could generate change was based on the Order of Food System Change criteria.
2. The food-related policy action’s **intent** to change the food system. This means that policy actions were evaluated based on their intent of changing the food system, regardless of the order of change that was achieved in practice.
3. The food-related policy action’s **potential** to change the food system. This means that the quality of the policy’s design or the level of investment in its implementation were not considered in the evaluation process. Instead, the evaluation was focussed on the ability of the policy action to change the food system had it achieved its full design and implementation potential.
4. The **substance** of the food-related policy action in changing the structure and/or operation of the food system. This means that, while characteristics such as whether a policy action is i) voluntary or mandatory, ii) monitored or not, iii) enforced or not, are important, these aspects are not considered for the evaluating policies in this analysis.
