# Supplementary material for: An analysis of the transformative potential of Australia’s national food policies and policy actions to promote healthy and sustainable food systems
Source: Public Health Nutr. 2024 Feb 20;27(1):e75. doi: 10.1017/S1368980024000478 (PMC10966843; doi:10.1017/S1368980024000478)
Supplement: Ribeiro de Melo et al. supplementary material 3 — Ribeiro de Melo et al. supplementary material [file S1368980024000478sup003.docx]

**Supplementary Table 2** Identification of Australian Federal Government food policies and evaluation of policy actions according to the orders of change they represent.

| **Responsible federal government departments** | **Food policies** | **Policy actions** | **Status** | **Order of change** | **Food system focus** |  |
| --- | --- | --- | --- | --- | --- | --- |
| **The Department of Health** | | | | | | |
| The Department of Health | *Get Up & Grow – Healthy eating and physical activity for early childhood* | The *Get Up & Grow – Healthy eating and physical activity for early childhood* is a collection which contains several resources to help parents and educators make sure children eat healthy foods and are physically active. | Available to the public since May 2010. Last updated in May 2023. | First-order change | Consumer behaviour |  |
| The Department of Health (through the National Health and Medical Research Council's - NHMRC) | *The Eat for Health website* | The Eat for Health website contains a collection of evidence-based guidelines for healthy eating (The Australian dietary guidelines) and the Nutrient Reference Values (NRVs) for Australia and New Zealand. The website also provides a range of consumer information resources for healthy eating and wellbeing, including information about the five food groups and the Nutrition calculator. | Available to the public. | First-order change | Consumer behaviour |  |
| The Department of Health | *feedAustralia* | The feedAustralia is a Department of Health’s initiative developed in partnership with the University of Newcastle, Hunter New England Population Health, and Healthy Australia. The feedAustralia App makes recipes and nutritional information available to parents of children enrolled in childcare services as well as other care providers at no cost. | Available to the public since 2017. | First-order change | Consumer behaviour |  |
| The Department of Health | *The National Healthy School Canteens (NHSC) project* | The NHSC project was funded by the Australian Government, as part of the Australian Better Health Initiative. The project has developed national guidance and training to help canteen managers make healthier food and drink choices for school canteens. Guidelines and resources are intended for use in school canteens across Australia and draw on existing national materials such as The Australian Guide to Healthy Eating and the 2013 Australian Dietary Guidelines, as well as state and territory resources. | Available to the public since 2008. Guidelines and resources are voluntary and do not provide endorsement of food or drink products. | Second-order change | Food environment |  |
| The Department of Health | *Healthy Food Partnership (HFP)*  The Healthy Food Partnership (the Partnership) is a voluntary collaborative forum that works with the Australian government, the public health sector and the food industry to encourage healthy eating among Australians; promote appropriate portion sizes; and enable food manufacturers to provide healthier choices. | The Health Star Rating (HSR) system: the HSR is a voluntary front-of-pack labelling system that rates the overall nutritional profile of packaged foods and assigns them a rating from ½ a star to 5 stars developed by the Australian governments in collaboration with industry, public health and consumer groups. | Active (voluntary). | First-order change | Food environment |  |
|  |  | The Voluntary Food Reformulation: the program aims to reduce sugar, sodium and saturated fat in processed and manufactured food and drinks to help improve population dietary intakes and help reduce diet-related chronic conditions. Foods for reformulation were chosen by a working group of experts from food industry, public health and government, and were selected due to being considered major contributors to sodium, saturated fat and/or sugar in Australians’ diets. Reformulation targets are based on the levels of risk nutrients in these foods, international reformulation targets, technical and safety data, and feasibility for reformulation. | Active (voluntary). | First-order change | Food supply chain |  |
|  |  | The Industry Guide to Voluntary Serving Size Reduction: the guide provides practical solutions to support the food industry to reduce the serving sizes for a number of discretionary foods and beverages in retail and out of home sectors. | Available to the public since April 2023. This guideline is voluntary and does not provide endorsement of serving sizes. | First-order change | Food supply chain |  |
|  |  | Provision of nutrition guidance to educate Australians on healthy eating. | No further information is available. | First-order change | Consumer behaviour |  |
| The Department of Health | *National Preventive Health Strategy (NPHS) (2021–2030)* | Nutrition and food action in Australia is guided by a specific national policy document | Policy achievement to be attained by 2030. | Second-order change | All |  |
|  |  | Nutrition information and guidance is translated and widely communicated for all health literacy level | Policy achievement to be achieved by 2030. | First-order change | Consumer behaviour |  |
|  |  | Structural and environmental barriers to breastfeeding are decreased through policy action | Policy achievement to be attained by 2030. | Second-order change | Food environment |  |
|  |  | Australian Dietary Guidelines are supported by a communication and social marketing strategy | Policy achievement to be attained by 2030. | First-order change | Consumer behaviour |  |
|  |  | Healthy eating is promoted through widespread multi-media education campaigns | Policy achievement to be attained by 2030. | First-order change | Consumer behaviour |  |
|  |  | Ongoing access to adequate and affordable healthy food options are available to all Australians, including older Australians | Policy achievement to be attained by 2030. | Second-order change | Food environment |  |
|  |  | A national policy document is developed to address food security in priority populations | Policy achievement to be attained by 2030. | Second-order change | All |  |
|  |  | Consumer choice is guided by the Health Star Rating system which is displayed on all multi- ingredient packaged food products | Policy achievement to be attained by 2030. | First-order change | Food environment |  |
|  |  | Children’s exposure to unhealthy food and drink marketing, branding and sponsorships is further restricted across all forms of media, including through digital media | Policy achievement to be attained by 2030. | Second-order change | Food environment |  |
|  |  | Reduced sugar, saturated fat, and sodium content of relevant packaged and processed foods through reformulation and serving size reduction | Policy achievement to be attained by 2030. | First-order change | Food supply chain |  |
|  |  | Consideration of tax reform for unhealthy foods | Policy achievement to be attained by 2030. | Second-order change | Food environment |  |
|  |  | Relevant guidelines and policies are regularly updated using the latest scientific evidence | Policy achievement to be attained by 2030. | First-order change | Consumer behaviour |  |
|  |  | Consumer choice is guided by energy and ingredient labelling on all packaged alcoholic products | Policy achievement to be attained by 2030. | First-order change | Consumer behaviour |  |
|  |  | The nutritional and health needs of priority populations are met through co-designed, community-based programs that are culturally appropriate | Policy achievement to be attained by 2030. | Second-order change | Consumer behaviour |  |
|  |  | Restricted promotion of unhealthy food and drinks at point of sale and at the end-of-aisle in prominent food retail environments, and increased promotion of healthy food options | Policy achievement to be attained by 2030. | Second-order change | Food environment |  |
| Commonwealth of Australia as represented by the Health Ministers Meeting 2022 | *National Obesity Strategy (NOS) (2022–2032)*  *Enabling Australians to eat well and be active* | Build a healthier food system that favours the production, processing and distribution of healthy food and drinks. Example actions: food trade agreements to support healthier food supply chains. | Policy strategy to be attained by 2032. | Second-order change | Food supply chain |  |
|  |  | Make sustainable healthy food and drinks more accessible. Example actions: land use planning schemes to protect high-quality agricultural land; or increase access to healthy and traditional foods through land use planning and policy. | Policy strategy to be attained by 2032. | Second-order change | Food environment |  |
|  |  | Explore and implement use of economic tools to shift consumer purchases towards healthier food and drink options. Example actions: policies to make farming, production, and manufacturing of healthy food attractive; financial incentives to promote healthy foods; or policy approaches that use price to reduce consumption of sugar-sweetened beverages and alcoholic beverages. | Policy strategy to be attained by 2032. | Second-order change | Food environment |  |
|  |  | Make processed food and drinks healthier. Example actions: strengthen reformulation targets; use of other policy or regulations to support healthy food choices (e.g., labelling, and compositional limits for adverse nutrients); improve the nutrient profile of unhealthy foods in food retail settings; or reduce the serving sizes of unhealthy foods. | Policy strategy to be attained by 2032. | First-order change | Food supply chain and food environment |  |
|  |  | Improve nutrition information to help consumers make healthier choices at the time of purchase. Example actions: **i**mprove the HSR; provision of information on unhealthy ingredients; or businesses to display energy content of standardised foods on menus and at point of sale. | Policy strategy to be attained by 2032. | First-order change | Food environment |  |
|  |  | Reduce exposure to unhealthy food and drink marketing. Example actions: promotion and sponsorship especially for children across all audio-visual media and on devices that appeal to children and in places visited by large numbers of people; protection of infant and families from the excess availability of marketing of breast milk substitutes and related products. | Policy strategy to be attained by 2032. | Second-order change | Food environment |  |
|  |  | Enable school and early childhood education and care settings to better support children and young people to be healthier**.** Example actions: nutrition education across the learning and education environment; whole-of-school practices to support healthy behaviours; build family and community partnerships to support learning outcomes; or create infrastructure to support healthy behaviours (community kitchens, food gardens, etc.) | Policy strategy to be attained by 2032. | Second-order change | Consumer behaviour |  |
|  |  | Enable workplaces to better support the health and wellbeing of their employees. Example actions: healthy food procurement and provision of foods especially in government institutions. | Policy strategy to be attained by 2032. | Second-order change | Food environment |  |
|  |  | Improve people’s knowledge, skills and confidence to lead active lives and to buy, prepare and enjoy healthy food and drinks. Example actions: provide information, education, and skill-building initiatives that promote and align with the ADGs; regularly update and promote Australian guidelines for healthy eating | Policy strategy to be attained by 2032. | First-order change | Consumer behaviour |  |
|  |  | Use social marketing to foster healthy social and cultural norms, reduce weight stigma and help people make healthy choices. Example actions: deliver social marketing and mass media campaigns; or partner with Aboriginal and Torres Strait Islander peoples and communities to deliver social marketing. | Policy strategy to be attained by 2032. | First-order change | Consumer behaviour |  |
|  |  | Enable parents, carers and families to optimize healthy child development and lifelong healthy habits for children and adolescents. Example actions: provide healthy eating guidance and support for parents after birth; or support women to breastfeed (Australian National Breastfeeding Strategy: 2019 and Beyond). | Policy strategy to be attained by 2032. | First-order change | Consumer behaviour |  |
|  |  | Engage and support young people to embed healthy behaviours as they transition to adulthood. | Policy strategy to be attained by 2032 | First-order change | Consumer behaviour |  |
|  |  | Engage and support local communities and organizations to develop and lead their own healthy eating and physical activity initiatives. Example actions: partner with young people to develop support to enhance healthy eating; low or no cost approaches to provide cooking education to young people with a focus on groups with low incomes. | Policy strategy to be attained by 2032 | First-order change | Consumer behaviour |  |
| Food Standards Australia New Zealand - FSANZ, the Food Regulatory System, and DAWE (responsible for the enforcement of the code) | *The Australia New Zealand Food Standards Code, 1991* | The standards in the Australia New Zealand Food Standards Code are legislative instruments under the Legislation Act 2003. It contains a series of food standards set out in four chapters: **Chapter 1 – General Food Standards, Chapter 2 - Food Product Standards, Chapter 3 – Food Safety Standards and Chapter 4 – Primary Production and Processing Standards** (see below). These legal standards are designed to ensure that food is safe for consumers; prevent misleading conduct associated with the sale of food; ensure that consumers to make informed decisions about the foods they purchase; and provide an effective regulatory framework within which the food industry can work efficiently. The Code is enforced by state and territory agencies and local councils for all food available for sale within their jurisdiction, including both imported and domestically produced food. The Department of Agriculture, Water, and the Environment (DAWE) is responsible for enforcing the Code at the border in relation to imported foods, which are subject to requirements under the Biosecurity Act 2015 and the Imported Food Control Act 1992. | In force. | Second-order change | Food environment |  |
| **The Department of Agriculture, Water and the Environment (DAWE)** | | | | | | |
| DAWE and DISER | *The National Landcare Program (NLP)*  The NLP is a nationwide program established in 1990, jointly delivered by DAWE and the Department of Industry, Science, Energy and Resources (DISER). The NLP contains a number of programs fund projects to impact regional and local levels in Australia. | The Regional Land Partnerships (RLP): the RLP are the largest component of the NLP, through which the government delivers nationwide projects to contribute to the recovery of threatened species and protection of ecological communities, reduce threats to globally important wetlands and world heritage sites, improve on-farm soil, biodiversity and vegetation, and increase the capacity of farms to adapt to climate change and evolving market demands. | Active. The government is investing funding from July 2018 to June 2023, to deliver national priorities at a regional and local levels. | Second-order change | Food supply chain |  |
|  |  | Environment Small Grants (ESG): the ESG has funded a total of 135 projects aimed to provide access to funding for local community projects to protect and conserve Australia’s water, plants and animals and the ecosystems. | Active. The government invested on phase two of the NLP, a funding which will be used in projects starting in June 2023. | First-order change | Food supply chain |  |
|  |  | Regional Natural Resource Management (NRM): the NRM organisations operate in 56 management units across Australia acting as delivery agents under the regional stream of the NLP. | Active. | First-order change | Food supply chain |  |
|  |  | Landcare Networks: these networks act as a conduit between local, regional and national levels for information sharing and coordination of the issues facing on-ground volunteers. | Active. The NLP phase two is continuing to support Landcare networks through allocated funding over 5 years to 2022-23. | First-order change | Food supply chain |  |
| DAWE | *Drought Policy* | The Australian Government works with all involved as they prepare for, manage, and recover from drought. Through the Future Drought Fund the AFG has invested in drought hubs, digital climate tools, business and risk management training for farmers, funding for organizations to build community drought resilience, trials to encourage the take up of drought resilience practices, and the development of drought resilience plans. | Active. | Second-order change | Food supply chain |  |
| DAWE | *The Australian Food Pact* | The Australian Food Pact is a multi-year commitment to reduce food waste by the businesses who grow, make and sell foods to reduce food waste. It is a voluntary agreement that encourages organizations to develop tailored food waste action plans focusing on preventing food waste, donating surplus good food, and supporting food chain transformation and innovation. | Active (voluntary agreement to halve food waste by 2030). | Second-order change | Food supply chain |  |
| DAWE | *The Agriculture Biodiversity Stewardship Package*  The Agriculture Stewardship Package is trialing with key partners (the Australian National University, the National Farmers’ Federation and the Natural Resource Management) market arrangements that encourage private investment in biodiversity and other sustainability opportunities in six pilot regions. | On-ground pilots based on a market approach-informed, such as the Carbon + Biodiversity Pilot and the Enhancing Remnant Vegetation Pilot, to reward landholders for undertaking carbon plantings, increasing biodiversity, and retaining and improving existing native vegetation on privately owned lands. | On trial (phase one). | First-order change | Food supply chain |  |
|  |  | The voluntary Australian Farm Biodiversity Certification Scheme: a voluntary certification scheme that enables consumers to identify farms that sustain biodiversity. It allows farmers to showcase their stewardship of the land to communities and markets. | Active (voluntary certification). | First-order change | Consumer behaviour |  |
|  |  | The National Stewardship Trading Platform: the platform integrates spatial information alongside buyer and seller to enable landholders to connect with buyers of biodiversity outcomes and kick-start private sector biodiversity markets. | Active. | First-order change | Food supply chain |  |
| **The Department of Industry, Science, Energy and Resources (DISER)** | | | | | | |
| DISER and Treasury | *Country of Origin Food Labelling Information Standard 2016 (Cth)* | It requires most food suitable for retail sale in Australia to carry country of origin information to help consumers make informed decisions about where food they buy is grown, produced, made, or packaged. This information may take the form of a text statement, or a text and graphic label known as a standard mark. | In force. | First-order change | Food environment |  |
| DISER | *Climate Active* | Climate Active is an ongoing partnership between the Australian Government and Australian businesses to drive voluntary climate action. A Climate Active certification is awarded to organizations (business operations), products, services, events, precincts and buildings that have credibly reached a state of carbon neutrality - based on an agreed emissions boundary for a specific certification type. | Active (voluntary partnership). | First-order change | Food supply chain |  |
| DISER (through the Clean Energy Regulator) | *The National Greenhouse and Energy Reporting Act 2007 (NGER Act) and the National Greenhouse and Energy Reporting (NGER) scheme* | The NGER National Greenhouse and Energy Reporting Act 2007 introduced a single national legislative framework, the Greenhouse and Energy Reporting (NGER) scheme, for reporting and disseminating company information about greenhouse gas emissions, energy production and energy consumption and other information specified under NGER legislation. Several legislative instruments sit under the NGER Act, providing greater detail about corporations'​​ obligations.  The Clean Energy Regulator administers the NGER Act, its legislative instruments, and related policies and processes. | In force. | Second-order change | Food supply chain |  |
| DISER (through the Clean Energy Regulator) and DAWE | *The Emissions Reduction Fund (ERF)* | The Emissions Reduction Fund offers landholders, communities, and businesses the opportunity to run projects in Australia that avoid the release of greenhouse gas emissions or remove and sequester carbon from the atmosphere. Participants, such as farmers, landholders, businesses, state governments or local councils, who want to reduce your carbon footprint, or are looking to participate in Australia’s carbon market register their projects and earn Australian carbon credit units (ACCUs). Each ACCU represents one ton of carbon dioxide equivalent (tCO2-e) emissions stored or avoided by a project. ACCUs can be sold to generate income, either to the Australian Government through a carbon abatement contract, or to companies and other private buyers in the secondary market. The ERF also includes a safeguard mechanism that requires Australia's largest greenhouse gas emitters to keep their net emissions below an emissions limit. | Enacted through the Carbon Credits Act 2011 and the Carbon Credits Rule 2015. | Second-order change | Food supply chain |  |
| **Treasury** | | | | | | |
| Australian Taxation Office (ATO) | *Goods and services tax (GST) - Section 38-2 of the Act 1999* | A supply of food is GST-free in accordance with section 38-2 of the A New Tax System (Goods and Services Tax) Act 1999 (the Act). The GST on foods establishes that basic foods such as fresh fruit, vegetables, bread, cereals, unflavoured milk, and cheese, are GST exempt, whereas prepared foods are not GST free. Foods that have been prepared and sold in a food service outlet such as a café or restaurant will have GST applied regardless of whether they comprise healthy (core) or unhealthy (discretionary) ingredients. | In force (the GST on foods was introduced in July 2000). | Second-order change | Food environment |  |
| **The Department of Foreign Affairs and Trade (DFAT)** | | | | | | |
| DFAT and DAWE | *Australia's free trade agreements (FTAs) for foods* | DFAT leads and coordinates Free Trade Agreements (FTAs) that are consistent with the World Trade Organization (WTO) guidelines on behalf of the Australian Government. FTAs reduce and eliminate certain barriers to international trade and investment and allow Australian exporters, importers, and producers to expand their business into foreign markets. These trade agreements also inform the regulations created by the DAWE for imported foods. DAWE works closely with DFAT to ensure the interests of agricultural industries are strongly represented in the development of Australian negotiating positions. | In force. From January 1983 to January 2022, Australia has concluded FTAs with 16 countries/ groups. | Second-order change | Food supply chain |  |
| **The Department of Social Services** | | | | | | |
| Department of Social Services (DSS) | *Emergency Relief National Coordination Plan*  The Australian Government is working with the emergency and food relief sectors to ensure support is available to vulnerable individuals and families experiencing financial crisis throughout these unprecedented times | Emergency Relief and Food Relief Support: through these services, the government aims to increase Emergency Relief providers’ access to a cost-effective supply of food items, across Australia. Under the Community Support Package, funding has been provided to several Commonwealth-funded Emergency Relief providers to (i) source, retain and increase workforce capacity; (ii) implement and extend home delivery services; and (iii) provide financial and material assistance to those who are at imminent risk of not being able to pay a bill. In addition, Commonwealth-funded Food Relief providers, such as the Foodbank Australia, SecondBite and OzHarvest, have been provided with funding to ensure food items are available to individuals and families in need across Australia. | Active. The government is investing funding over four and half years to 30 June 2023 for Emergency Relief and Food Relief Support services. | First-order change | Food supply chain |  |
|  |  | Supporting service providers in responding to the coronavirus outbreak: the Australian Government is supporting service providers, such as charities and other community organizations, in responding to the coronavirus outbreak, as well as directing funding for Emergency Relief and Food Relief support. | Implemented. On 29 March 2020, the Australian Government announced funding to support charities and other community organizations in responding to the coronavirus outbreak. | First-order change | Food supply chain |  |
|  |  | The National Coordination Plan: the national coordination plan supports the identification and analysis of local, state and sector issues and needs, and oversees the implementation of emergency and food relief across the country. The Australian Government works with the National Coordination Group to monitor the impact of the coronavirus pandemic and allocate further funding where it is most needed. | Active. | First-order change | Food supply chain |  |
| **The Department of Infrastructure, Transport, Regional Development and Communications** | | | | | | |
| Department of Infrastructure, Transport, Regional Development and Communications (through the Australian Communications and Media Authority) | *Broadcasting Services Act 1992 (the Act)* | The Broadcasting Services Act 1992 (the Act) provides a regulatory environment for the Australia’s broadcasting, datacasting, and the online content service industries. It contains one provision that specifically restricts unhealthy food advertising during children programming: *“An advertisement for a food product may not contain any misleading or incorrect information about the nutritional value of that product.”* Some of the other general rules on advertising are also relevant to foods, such as the ones that ban that advertisements encourage children to ask their parents to buy the advertised item; those that present “premiums” in ways that are more than incidental; or those that use a popular personality to promote a product. | In force. | Second-order change | Food environment |  |
| The Department of Infrastructure, Transport, Regional Development and Communications and DAWE | *Regional Airports Program (RAP)* | A program that facilitates improved delivery of essential goods and services such as food supplies, health care and passenger air services. Through the Remote Air Services Subsidy (RASS) Scheme, it subsidizes through competitive grants a regular air transport service for the carriage of passengers and goods such as educational materials, medicines, fresh foods and other urgent supplies to communities in remote and isolated areas of Australia. | Active. The Australian Government committed funding over four years to 2022–23 to the Regional Airports Program. | Second-order change | Food supply chain |  |
| Department of Infrastructure, Transport, Regional Development and Communications (through the National Water Grid Authority) | *Australia’s National Water Grid* | The Australia’s National Water Grid is a series of region-specific water storage and distribution solutions to secure predictable supplies of water now and into the future. Through a range of planning, science and construction projects, the national water grid is promoting agricultural opportunities in new regions and increasing water security in established agricultural areas. | Active. | Second-order change | Food supply chain |  |
| The Department of Infrastructure, Transport, Regional Development and Communications and DAWE | *Inland Rail* | The Inland Rail is an infrastructure project, and a vital component of the National Freight and Supply Chain Strategy (the Strategy), that will facilitate the transporting the food from agricultural land Australia produces to domestic and international communities. The over 1,700-kilometre new Inland Rail freight route will connect the north to south, Brisbane to Melbourne, in under 24 hours, transporting products across large distances more quickly, in greater volumes and at a reduced cost. | Underway | Second-order change | Food supply chain |  |
| **The Department of Education, Skills, and Employment (DESE)** | | | | | | |
| The Australian Curriculum, Assessment and Reporting Authority | *The Australian National Curriculum* | The Australian Curriculum addresses student learning regarding food and nutrition through the Health and Physical Education subject. Students start to learn about food and nutrition from years 1-10, and the connection between food and wellbeing is presented throughout the different stages of schooling. | Implemented (ongoing) | First-order change | Consumer behaviour |  |
